# Lithium-plasmon-based low-powered dynamic color display

**DOI:** 10.1093/nsr/nwac120

**Published:** 2022-06-23

**Authors:** Jie Liang, Yan Jin, Huiling Yu, Xinjie Chen, Lin Zhou, Pengcheng Huo, Ye Zhang, Haiyang Ma, Yi Jiang, Bin Zhu, Ting Xu, Hui Liu, Shining Zhu, Jia Zhu

**Affiliations:** National Laboratory of Solid State Microstructures, College of Engineering and Applied Sciences, School of Physics, Key Laboratory of Intelligent Optical Sensing and Manipulation, Ministry of Education, Jiangsu Key Laboratory of Artificial Functional Materials, Nanjing University, Nanjing 210093, China; National Laboratory of Solid State Microstructures, College of Engineering and Applied Sciences, School of Physics, Key Laboratory of Intelligent Optical Sensing and Manipulation, Ministry of Education, Jiangsu Key Laboratory of Artificial Functional Materials, Nanjing University, Nanjing 210093, China; National Laboratory of Solid State Microstructures, College of Engineering and Applied Sciences, School of Physics, Key Laboratory of Intelligent Optical Sensing and Manipulation, Ministry of Education, Jiangsu Key Laboratory of Artificial Functional Materials, Nanjing University, Nanjing 210093, China; National Laboratory of Solid State Microstructures, College of Engineering and Applied Sciences, School of Physics, Key Laboratory of Intelligent Optical Sensing and Manipulation, Ministry of Education, Jiangsu Key Laboratory of Artificial Functional Materials, Nanjing University, Nanjing 210093, China; National Laboratory of Solid State Microstructures, College of Engineering and Applied Sciences, School of Physics, Key Laboratory of Intelligent Optical Sensing and Manipulation, Ministry of Education, Jiangsu Key Laboratory of Artificial Functional Materials, Nanjing University, Nanjing 210093, China; National Laboratory of Solid State Microstructures, College of Engineering and Applied Sciences, School of Physics, Key Laboratory of Intelligent Optical Sensing and Manipulation, Ministry of Education, Jiangsu Key Laboratory of Artificial Functional Materials, Nanjing University, Nanjing 210093, China; National Laboratory of Solid State Microstructures, College of Engineering and Applied Sciences, School of Physics, Key Laboratory of Intelligent Optical Sensing and Manipulation, Ministry of Education, Jiangsu Key Laboratory of Artificial Functional Materials, Nanjing University, Nanjing 210093, China; National Laboratory of Solid State Microstructures, College of Engineering and Applied Sciences, School of Physics, Key Laboratory of Intelligent Optical Sensing and Manipulation, Ministry of Education, Jiangsu Key Laboratory of Artificial Functional Materials, Nanjing University, Nanjing 210093, China; National Laboratory of Solid State Microstructures, College of Engineering and Applied Sciences, School of Physics, Key Laboratory of Intelligent Optical Sensing and Manipulation, Ministry of Education, Jiangsu Key Laboratory of Artificial Functional Materials, Nanjing University, Nanjing 210093, China; National Laboratory of Solid State Microstructures, College of Engineering and Applied Sciences, School of Physics, Key Laboratory of Intelligent Optical Sensing and Manipulation, Ministry of Education, Jiangsu Key Laboratory of Artificial Functional Materials, Nanjing University, Nanjing 210093, China; National Laboratory of Solid State Microstructures, College of Engineering and Applied Sciences, School of Physics, Key Laboratory of Intelligent Optical Sensing and Manipulation, Ministry of Education, Jiangsu Key Laboratory of Artificial Functional Materials, Nanjing University, Nanjing 210093, China; National Laboratory of Solid State Microstructures, College of Engineering and Applied Sciences, School of Physics, Key Laboratory of Intelligent Optical Sensing and Manipulation, Ministry of Education, Jiangsu Key Laboratory of Artificial Functional Materials, Nanjing University, Nanjing 210093, China; National Laboratory of Solid State Microstructures, College of Engineering and Applied Sciences, School of Physics, Key Laboratory of Intelligent Optical Sensing and Manipulation, Ministry of Education, Jiangsu Key Laboratory of Artificial Functional Materials, Nanjing University, Nanjing 210093, China; National Laboratory of Solid State Microstructures, College of Engineering and Applied Sciences, School of Physics, Key Laboratory of Intelligent Optical Sensing and Manipulation, Ministry of Education, Jiangsu Key Laboratory of Artificial Functional Materials, Nanjing University, Nanjing 210093, China

**Keywords:** lithium plasmon, dynamic display, lithium metal battery, low energy consumption, lithium nanoparticle

## Abstract

Display and power supply have been two essential and independent cornerstones of modern electronics. Here, we report a lithium-plasmon-based low-powered dynamic color display with intrinsic dual functionality (plasmonic display and energy recycling unit) which is a result of the electric-field-driven transformation of nanostructured lithium metals. Dynamic color displays are enabled by plasmonic transformation through electrodeposition (electrostripping) of lithium metals during the charging (discharging) process, while the consumed energy for coloring can be retrieved in the inverse process respectively. Energy recycling of lithium metals brings energy consumption down to 0.390 mW cm^−2^ (0.105 mW cm^−2^) for the active (static) coloration state of a proof-of-concept display/battery device, which approaches nearly-zero-energy-consumption in the near-100%-energy-efficiency limit of commercial lithium batteries. Combining the subwavelength feature of plasmonics with effective energy recycling, the lithium-plasmon-based dynamic display offers a promising route towards next-generation integrated photonic devices, with the intriguing advantages of low energy consumption, a small footprint and high resolution.

## Teaser

Low-powered dynamic color display based on lithium-plasmon was for the first time reported with intrinsic dual-functions, the plasmonic display and energy recycling unit, through electric-driven transformation of nanostructured lithium metals.

## INTRODUCTION

Display and power supply are two essential and independent building blocks that support modern electronics [1–5]. Driven by premier applications ranging from portable mobile electronics to large-scale indoor and/or outdoor billboards, displays and batteries have seen significant advancements in the past. For next-generation mobile devices, both energy-efficient display modules, surpassing organic light-emitting display technologies (>10 mW cm^−2^), and high-energy-density portable batteries are urgently needed [6–12]. While displays and batteries need to work seamlessly, as displays consume a significant proportion (up to 68%) of power for electronics [13–16], the development paths of the two fields have never converged thus far.

Alkali metals are attracting significant attention at the intersection of plasmonics and energy storage [17–19]. On the one hand, alkali metal is emerging as a low-loss and active plasmonic material [19–22]. Plasmonics, with the unique capability of focusing light into a subwavelength scale, offers a promising solution for high-resolution displays [23–28], with features prominently compared with other structural color materials like all-dielectric metasurfaces, semiconductors and phase change materials [29–35]. In addition, external electrical (such as nanostructured noble metal deposition [36]), chemical or other stimuli can be more effective at enabling dynamic displays with respect to dielectric-based nanophotonic counterparts [37–46]. On the other hand, lithium (Li), as the lightest metal, has long been regarded as the holy grail of high-energy-density anode materials [10,12], with a high specific capacity (3860 mAh g^−1^) and the lowest electrochemical potential (−3.04 V versus the standard hydrogen electrode).

Here, for the first time, we demonstrate a Li-metal-based low-powered dynamic plasmonic color display, which is simultaneously a nanostructured anode of a Li metal battery, with the inherit advantages of dynamic tunability and extremely low energy consumption. During the charging process, Li metal nucleates and grows on a pre-patterned substrate, resulting in the generation and tuning of plasmonic colors. During the discharging process, Li metal strips off from the substrate, thus leading to the erasure of the color.

The energy storage feature of Li metal enables effective energy recycling and reduces the overall energy consumption to 0.390 mW cm^−2^ for the active coloration state and 0.105 mW cm^−2^ for the static coloration state of a proof-of-concept display/battery device, vital for energy-efficient display technologies. The dual functionality of display and power supply enables a low-powered display device, in which one charged pixel can power another one to release its energy, and vice versa, during repeated cycling. Our results may offer a unique opportunity to develop a nano-scaled integrated platform for energy storage and information display.

## RESULTS AND DISCUSSION

### The schematic of the low-powered dynamic plasmonic color display

**Figure 1.**
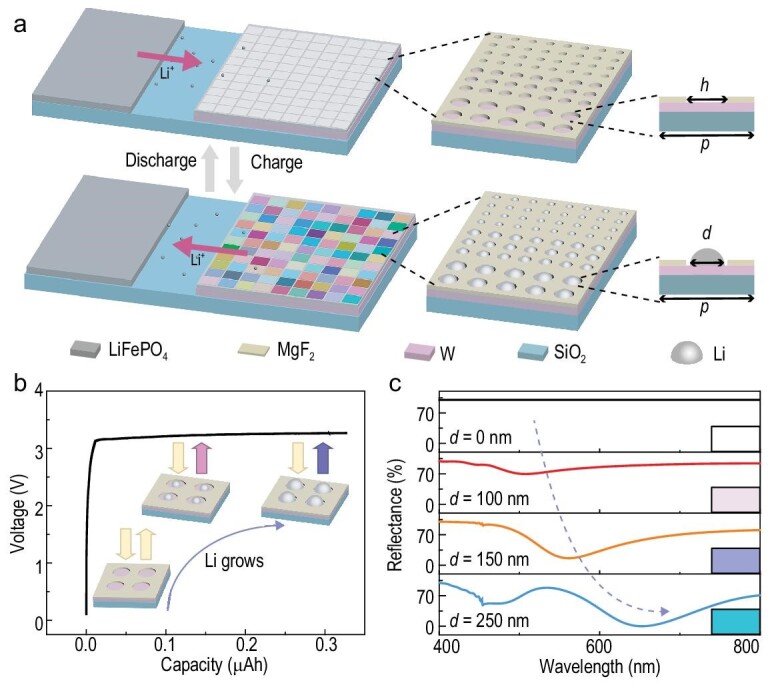
Schematic and illustration of the low-powered dynamic plasmonic color display. (a) The schematic and mechanism of operation. The display panel, which serves as the anode of the battery, is composed of silica (SiO_2_) substrate, tungsten (W) conductive film (100 nm thick) and a magnesium fluoride (MgF_2_) insulating layer (30 nm thick) with periodic holes under the colorless ‘off’ state. Each pixel is composed of periodic holes with a given lateral period (*p*) and diameter (*h*). During the charging (electrodeposition) process, Li nanoparticles with diameter (*d*) generate colors as the ‘on’ state. These colors can be erased during the discharging process once Li nanoparticles are stripped off. (b) The voltage profile of the dynamic system as a function of charging capacity during the charging process. Insets show the evolution of Li structures and reflection colors. (c) The simulated reflection spectra as functions of the diameter (*d*) of Li metal nanoparticles (with constant lateral period *p* = 320 nm) and the corresponding colors (on the right).

The schematic of the low-powered dynamic color display of plasmonic lithium metals is illustrated in Fig. 1a. It behaves as a planar Li metal battery simultaneously (see Methods and Supplementary Fig. 1 for fabrication details; see the optical photograph and scanning electron microscope (SEM) images of the integrated display unit in Supplementary Fig. 2). Since the display panel is also a battery, it is composed of a Li source (LiFePO_4_) as the cathode and a perforated magnesium fluoride (MgF_2_) film on tungsten (W) as the anode template, with liquid electrolyte as the ambience. Note that the pre-patterned anode template with the periodically perforated MgF_2_ film (*p* = period, *h* = the diameter of the hole; see magnified schematic in the top panel of Fig. 1a) is originally at the colorless ‘off’ state.

Serving as both the core element of color pixels for display, and as the anode for the battery, the microstructure of Li nanoparticles is electrically programmable by the electrochemical reactions. During the charging process, as a charging current is applied, Li ions from the cathode (LiFePO_4_) migrate towards the pre-patterned anode template. Once these Li ions are reduced at the target sites, Li metal nucleates and electrodeposits into the pre-patterned holes of the MgF_2_ film, forming the well-defined nano-hemispheres (*d* = diameter, see schematic in the bottom panel of Fig. 1a) that serve as the reflective pixel units of the colorful ‘on’ state. Note that the electric-field-driven redox reaction of the battery is reversible. During the discharging process, the deposited Li metal nanoparticles can be removed from the holes of the anode and reset the colorful display back to a colorless ‘off’ state.

The correlation between the optical parameters for the color display (the reflection spectra in visible and corresponding colors) and the electrochemical parameters for the battery (the applied voltage and electric capacity) is established (Fig. 1a–c). Figure 1b depicts the battery voltage and qualitative structural evolution profiles of Li metal nanoparticles as a function of the capacity (note: capacity is defined as the product of current density and time). Li metal nanoparticles grow as the charging capacity increases, which changes the reflective spectral response, resulting in the real-time plasmonic coloration (schematically depicted in the insets in Fig. 1b). Based on systematic simulations (see Methods and Supplementary Fig. 3 for details), Fig. 1c further illustrates the quantitative correlation between the evolved Li nanoparticle sizes (referring to the charging capacity) and reflective spectral responses, as well as the corresponding reflective colors. Detailed theoretical analyses on the absorption cross section (Supplementary Fig. 4) and electric field distribution profiles (Supplementary Fig. 5) reveal that localized surface plasmon resonance (LSPR) of the as-grown isolated Li nanoparticles, instead of the surface diffraction modes of periodic structures, plays a crucial role in plasmonic color generation.

### Electrochemical tunability of Li-metal-based plasmonic multi-colors

**Figure 2.**
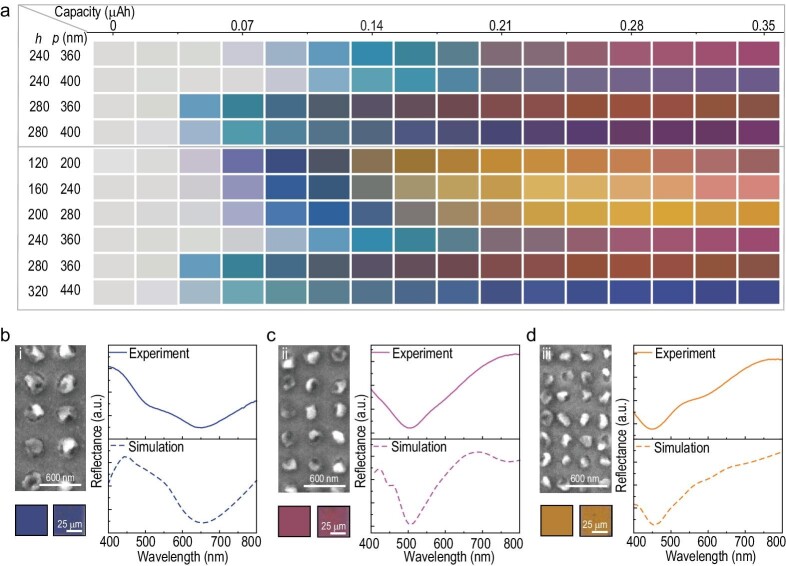
Structural dependence of the Li-based plasmonic color display. (a) The capacity-dependent structural color panels derived from microscopic images of Li patterns with different lateral periods *(p)* and hole diameters *(h)* during coloration. (b–d) Measured and simulated reflectance spectra of three representative structural colors as well as relevant SEM and color patches (the left colors are derived from the right microscope image): (i) *p* = 440 nm, *d* = 280 nm, *h* = 280 nm; (ii) *p* = 360 nm, *d* = 240 nm, *h* = 180 nm; and (iii) *p* = 280 nm, *d* = 160 nm, *h* = 160 nm, respectively.

A more comprehensive demonstration of the electrochemical tunability of Li plasmonic color is experimentally illustrated in Fig. 2a. To precisely tune the structural colors, periodic nanoholes (*p* = period, *h* = hole diameter) were prepared on the electrode by electron beam lithography (EBL) for the electric-field-driven Li metal deposition. In order to render a broad-range palette of colors, stepwise tuning of the charging capacity was employed for a gradual, incremental change in Li nanoparticle size (*d*). The pixel colors shown in Fig. 2a are functions of geometry parameters (*p*: 200–440 nm, *h*: 120–320 nm) and deposition capacity (0–0.35 μAh), which are derived from measured optical images of the structured anodes by arbitrarily getting the RGB (red, green, blue) values using the color picker in Photoshop (see Supplementary Fig. 8). The top part of Fig. 2a demonstrates that at the beginning depositing state, *h* determines the color because *h* affects the size of the Li nanoparticle (*d*), which plays a crucial role in the LSPR mode color generation, while at the later charging state, *p* also affects the color because as Li particles grow the electromagnetic field of each nanoparticle overlaps and the impact of *p* cannot be ignored (more discussions are shown in Supplementary Fig. 6). One may find that plasmonic colors of a wide color gamut can be enabled with patterned Li nanoparticles by fine-tuning particle sizes and charging capacity (Fig. 2a), revealing the capability of multi-color dynamic control.

We further exhibit three representative plasmonic colors of different Li patterns by running the electrodeposition experiments for a fixed charging capacity of ∼0.33 μAh, as shown in Fig. 2b–d. In these pixels, the diameters of deposited Li nanoparticles are 280 nm, 240 nm and 160 nm (measured from *ex-situ* SEM images i, ii and iii of Fig. 2b–d) for blue, red and yellow colors, with the measured reflectance dips at 650 nm, 500 nm and 450 nm, respectively. The simulated reflection spectra of the Li patterns (with parameters retrieved from the *ex-situ* SEM images) agree well with the measured ones, which confirms the plasmon-enabled color generation.

### Dynamic plasmonic color displays

**Figure 3.**
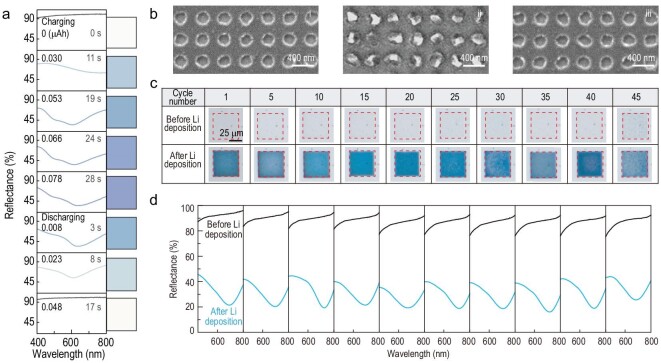
Dynamic plasmonic color displays and cycling performance. (a) A complete generating/erasing cycle of blue color characterized by the measured reflectance spectra and derived colors. (b) SEM images of the blue color display panel (*p* = 400 nm, *h* = 200 nm) at the (i) original, (ii) color generation and (iii) color erasure states during one cycle. (c and d) Cycling performance of a dynamic blue coloration: (c) optical images, (d) the correlated reflectance spectra.

As the built-in structural transformation and reflectance tunability are confirmed, we further demonstrate the capability of electric-field-driven dynamic coloration. To evaluate the cycling performance of the structural color display, we perform *in-situ* measurements on reflectance spectra of the representative blue color (*p* = 400 nm, *h* = 200 nm), as shown in Fig. 3. During the *in-situ* charging/discharging experiment, the measured reflectance spectra gradually evolve from the initial colorless state (with nearly 100% reflectance in the visible regime) to the state of different shades of blue colors (with reflection dip at around 600 nm). It finally returns to the original state, with nearly 100% reflectance in the visible regime, after a complete cycle (Fig. 3a). The corresponding *ex-situ* morphologies of the display panel during the original, color generation and color erased states are shown in Fig. 3b. After the charging process with the generated structural color, Li nanoparticles are precisely deposited into the holes (Fig. 3b(ii)). As the color erasing process is terminated in the discharging process, the deposited Li nanoparticles are fully removed with the empty holes recovered (Fig. 3b(iii)), which validates the reversible structural transformation and thus dynamic colors of Li nanoparticles, as suggested in Fig. 1.

To evaluate the cycling performance of the dynamic plasmonic color display, we test the reflectance spectra of the ‘on/off’ coloration for at least 45 cycles via repeatable charging and discharging processes, as shown in Fig. 3c and d. The blue color is well maintained at the color generation states (or ‘on’ state after Li deposition) and fully removed at the ‘off’ states (before Li deposition) during long cycling (Fig. 3c). The considerable optical contrast (>50% intensity of reflectance dip) and wavelength of the reflectance dip (∼700 nm) at coloration states are repeatable during cycles (Fig. 3d). The overall cycling performance makes it a promising candidate for the dynamic color display.

**Figure 4.**
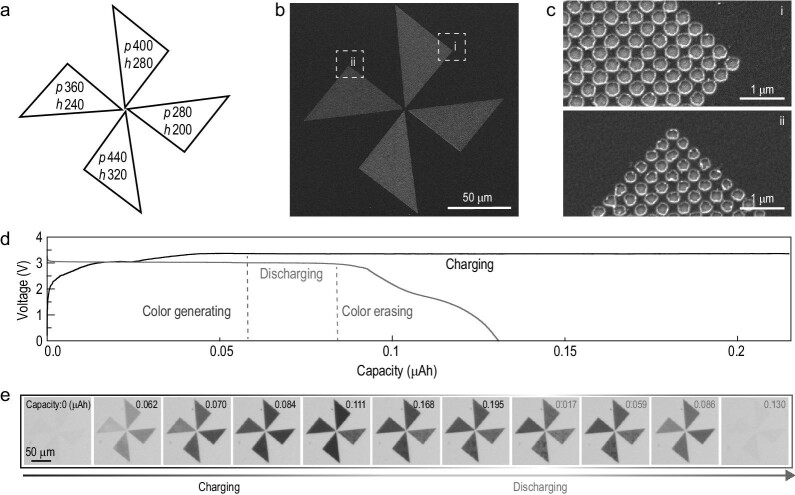
Plasmonic animations of a chromatic windmill pattern. (a) Design of a color display panel—a windmill pattern. (b and c) Overview and enlarged SEM images of the windmill pattern. (d) The battery voltage profile during charging and discharging processes. (e) The dynamic process of the plasmonic animation during charging and discharging processes for color generating and erasing, corresponding to the charging capacity and discharging capacity (labeled upper numbers, μAh).

To further unravel the dynamic tunability of multiple structural colors, we have designed a plasmonic animation of a chromatic micro-windmill showing full cycling, with schematic and SEM images shown in Fig. 4a–c, respectively. The pattern geometries of the four regions are carefully designed with geometry parameters labeled in Fig. 4a. The charging and discharging current densities of ∼0.4 mA cm^−2^ and 0.2 mA cm^−2^ are applied respectively (see Fig. 4d for the voltage profile). Figure 4e illustrates the real-time animation of the chromatic windmill over the charging/discharging capacity. A variety of color generation takes place within 0.062 μAh. Subsequently, the color of the windmill changes as a function of the charge capacity (until 0.195 μAh). During the discharging process, the colors of the windmill are completely erased after a discharging capacity of 0.130 μAh. Furthermore, an electric-field-driven micro-animation of a flying butterfly is demonstrated as well (Movie S1 and Supplementary Fig. 10). It indicates that this dynamic color display can be well programmed by the applied electrical signals, exhibiting compatibility with versatile smart electronic devices.

### Low-powered dynamic plasmonic color display

**Figure 5.**
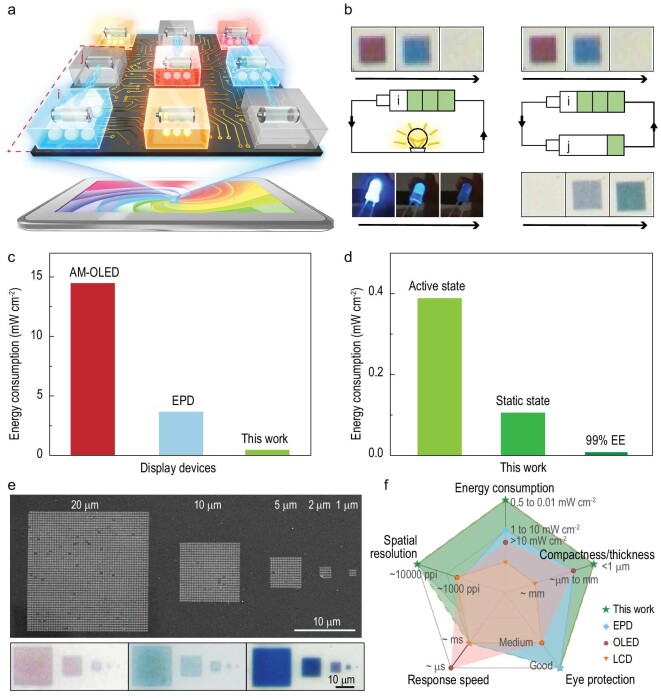
Low-powered dynamic plasmonic color display. (a) The operation schematic of the low-powered dynamic plasmonic color display: the display contains many pixels with some pixels erasing color and some generating color, so the color-erasing pixels with stored energy (e.g. pixel i) can recharge and power other pixels (e.g. pixel j) for color generation. (b) The dual functionality, i.e. power supply and information display, of the device. (c and d) A comparison of the energy consumption of different display devices [37,47]. The energy consumption of the proposed proof-of-concept device is evaluated as ∼0.390 mW cm^−2^ at the active coloration state for around 56.70% energy efficiency (EE) (even lower to ∼0.009 mW cm^−2^ for 99.00% EE in the future), and ∼0.105 mW cm^−2^ at the static coloration state. (e) SEM and optical images of pixels with different sizes (20 μm, 10 μm, 5 μm, 2 μm, 1 μm) for different colors. (f) A comparison of different display technologies in spatial resolution, energy consumption, response speed, compactness/thickness and eye protection [37,47–50].

Finally, we demonstrate the low-powered nature of the proposed Li-plasmon-based dynamic display device with rational electronic designs such as employing one pixel in a panel to charge another and vice versa. One of these low-powered strategies is shown in Fig. 5a. The proposed proof-of-concept device can consist of a large number of plasmonic color pixels (or mini-batteries, represented by the colored boxes in Fig. 5a), some of which are operating at the color erasing states (in the discharging process) while others are at color generating states (in the charging process). In detail, for an arbitrary plasmonic pixel (noted as i), once it experiences the discharging process, the output power can light up the LED until the stored energy (see the lower photos of the left panel in Fig. 5b) is exhausted, while the electrically driven structure transformation can simultaneously result in a capacity-dependent color evolution (see the upper photos of the left panel in Fig. 5b), revealing each pixel's dual functionality of information and energy (details are shown in the Supplementary Fig. 14).

To further optimize the energy recycling of the overall display panel and realize a low-powered display, we take an arbitrary pair of pixels for demonstration. As depicted in the right panel of Fig. 5b, the colored pixel (i) with stored energy can recharge and power the other pixel (j) without external power input. During these processes, each pixel (i and j) can output an independent time-resolved color (as information carrier) while energy consumed by each pixel will be recycled by its counterpart via the reversed electrochemical process (see Supplementary Fig. 15 for experimental details). Note that, the two-versus-one-pixel cycling configuration employed in Supplementary Fig. 15 is just a simplified case to compensate for the overpotential mismatch during an entire cycling process. However, it is definitely unnecessary provided that more dedicated DC-DC voltage conversion strategies (widely employed in commercial electronic devices) are applied to enable a one-versus-one-pixel configuration.

With the clearly identified dual functionality of these independent pixels (they are both the basic elements of color information and micro-power-suppliers for the display), one can quantitatively evaluate the energy consumption of the integrated display device. A direct comparison of the energy consumption of our work with representative commercial display devices is depicted in Fig. 5c. Taking the average energy efficiency (∼56.70%) of our prototype device, for example, the specific energy consumption is ∼0.390 mW cm^−2^ without optimization, which is at least one order of magnitude lower than the commercial active matrix organic light-emitting diode (AMOLED) (at the brightness of 160 cd cm^−2^ and contrast ratio of 10 000 : 1) or several times lower than the electronic paper display (EPD) system [37,47]. Moreover, if the display is operated at a static coloration state, the specific energy consumption can be even three times lower (0.105 mW cm^−2^, see Methods for detailed explanations). In addition, aiming at the intersection of plasmonics and energy storage, this technology will benefit from promising advancements in both fields. For instance, once the energy efficiency of the Li-metal-battery system reaches the same level as mainstream commercial batteries (∼99.00%), the overall energy consumption can be reduced down to 0.009 mW cm^−2^, which suggests a power reduction by about three orders of magnitude compared with the commercial AMOLED (Fig. 5d).

Finally, the pixel-scaling limit of the proposed lithium plasmonic device is experimentally evaluated and shown in Fig. 5e and Supplementary Fig. 16. Full-color pixels with different sizes (decreasing from 20 μm to 1 μm) and different colors in a square shape can be observed using a conventional optical microscope, revealing that the full-color pixel size can be as low as 1 μm even in the complicated liquid surroundings (which is approximately less than one half of that reported for the metasurface counterpart [8]). This enables the pronounced high spatial resolution of the electrically dynamic color display, one order of magnitude higher than the resolution of current displays (∼1000 PPI, e.g. LCD, LED and EPD), even in the rather complicated liquid environment [48,50].

As a systematic evaluation of the proposed Li-plasmon-based dynamic display, in the radar chart in Fig. 5f we compare this work with different display technologies using five representative parameters: spatial resolution, energy consumption, response speed, compactness/thickness and eye protection. Benefiting from the light field manipulation beyond the diffraction limit and the energy storage battery of Li metals, this work shows much lower energy consumption, much higher spatial resolution, higher compactness, considerable modulation speed and good eye protection for the full-color display [37,47–50]. Thus, this Li-metal-based plasmonic color display shows the best overall performance, opening up a promising direction for future high-performance display technology, especially in augmented reality applications.

## CONCLUSION

In summary, we demonstrate a low-powered Li-metal-based plasmonic device, with the dual functionality of an electric-field-driven color display and energy recycling unit. Li metal nucleates, grows and strips off from the precisely patterned anode inside the Li-metal battery, leading to the generation, change and erasure of plasmonic colors on the dynamic color display. The plasmonic feature of Li metals gives the display device rather high spatial resolution while the energy storage feature of the Li metals essentially lowers the energy consumption of the dynamic plasmonic color display to 0.390 mW cm^−2^ for the dynamic and 0.105 mW cm^−2^ for the static coloration. The technique of using Li metal plasmonics as both an information carrier (plasmonic material) and an energy carrier (battery anode) will benefit from the advancement of both fields to provide a promising strategy towards energy-efficient and high-resolution integrated photonic platforms.

## METHODS

### Fabrication of the Li-metal-based dynamic plasmonic color display system

The Li-metal-based dynamic plasmonic color display device is a planar anode-free Li-metal battery. It consists of three parts: nanostructured anode, liquid electrolyte and cathode. The anode template was fabricated through EBL using the steps demonstrated in Supplementary Fig. 1. A detailed description of this fabrication is available in the Supplementary Data.

### Characterization

The optical color images and dynamic animation were obtained using a brightfield reflection microscope (Nikon) illuminated by a light source (Energetiq Laser-Driven Light Source, EQ-99). A digital charge-coupled device (CCD) camera (Allied-Vision Prosilica GT2450C) was used to capture the color micrographs with a 20 × (NA ¼ 0.4) objective. The optical reflection spectra were measured in reflection mode using a microspectrometer (CRAIC) with unpolarized light incident perpendicularly to the sample surface. The measured reflectance spectra were normalized with respect to a bare region (MgF_2_/W/SiO_2_ flat film soaking in the electrolyte) next to the periodic hole structures.

An electrochemical workstation (Biologic SP-20) was used to control the Li nanoparticle deposition/stripping during the charging and discharging processes through galvanostatic cycling, and to measure the time-dependent potentials. The morphologies and structures of the dynamic display panels at the beginning, coloration and color-erasing states were characterized by SEM (Tescan Mira3). For the characterization of Li morphologies after the electrochemical deposition/stripping, the display device was disassembled in an Ar-filled glove box after optical reflectance measurement, then rinsed with fresh diethyl carbonate and dried. Display panels were mounted onto SEM stages and sealed in Ar-filled transfer vessels for immediate SEM observation to avoid oxidation of Li metal.

### Numerical simulations

The finite-difference time-domain method was used to calculate the reflection spectrum. For simplicity, a periodic boundary condition, and a plane wave as the excitation source, were used for the modeling. The material parameters of Li metal and W were from the Palik data, and the refractive index of the SiO_2_ substrate, MgF_2_ insulating layer and electrolyte were set as 1.45, 1.38 and 1.4, respectively. In order to simplify the simulations, the morphology of deposited Li metal was set as hemispheres with a diameter (*d*). The period (*p*) was set between 200 nm and 440 nm. The thicknesses of the SiO_2_ substrate, W conductive layer, MgF_2_ insulating layer and electrolyte layer were 1 mm, 100 nm, 30 nm and 1 μm, respectively.

### Chromaticity calculation

We calculated the color of the deposited Li metal hemispheres according to the International Commission on Illumination (CIE) ‘standard observer’ functions, based on human data. The details are available in the Supplementary Data.

### Energy consumption calculation

The energy consumption of our plasmonic display during the display is calculated with the following formulation:

\begin{eqnarray*} P\, = \,\overline{{U}_C}{I}_C\,(1\, - \,EE). \end{eqnarray*}

Here, $\overline {{U}_c} $ is the average charge voltage, *I_c_* is the charge current and *EE* is energy efficiency, which is the ratio of the discharged energy to the charged energy. The detailed calculations are shown in the Supplementary Data.

## Supplementary Material

nwac120_Supplemental_Files
