# Supplementary material for: Lithium-plasmon-based low-powered dynamic color display
Source: Natl Sci Rev. 2022 Jun 23;10(1):nwac120. doi: 10.1093/nsr/nwac120 (PMC9942666; doi:10.1093/nsr/nwac120)
Supplement: nwac120_Supplemental_Files [file nwac120_supplemental_files.zip › supplementary_data.docx]

**Supplementary information for：**

**Lithium plasmon based low-powered dynamic color display**

**AUTHORS:** Jie Liang^1, †^, Yan Jin^1, †^, Huiling Yu^1^, Xinjie Chen^1^, Lin Zhou^1,^ *, Pengcheng Huo^1^, Ye Zhang^1^, Haiyang Ma^1^, Yi Jiang^1^, Bin Zhu^1^, Ting Xu^1^, Hui Liu^1^, Shining Zhu^1^ and Jia Zhu^1,^ *

**AFFILIATIONS:**

^1^ National Laboratory of Solid State Microstructures, College of Engineering and Applied Sciences, School of Physics, Key Laboratory of Intelligent Optical Sensing and Manipulation, Ministry of Education, Jiangsu Key Laboratory of Artificial Functional Materials, Nanjing University, Nanjing 210093, P. R. China.

*Correspondence to: linzhou@nju.edu.cn, jiazhu@nju.edu.cn

^†^ These authors contributed equally to this work.

**CONTENTS:**

S. I. Methods.

S. II. Rational designs for electrodeposition and structural transformation.

S. III. Optical modes of the lithium plasmonic nanostructures.

S. IV. Plasmonic color generation in the charging/discharging process.

S. V. Electrochemical properties of the full-cycle display process.

S. VI. On-chip demonstration of the low-powered plasmonic color display panel.

References

**S. I. Methods**

1. **Fabrication of the Li metal-based dynamic plasmonic color display system.** The Li metal-based dynamic plasmonic color display device is a planar anode-free Li metal battery. It consists of three parts: nanostructured anode, liquid electrolyte and cathode. The anode template was fabricated through EBL with the following steps as demonstrated in Supplementary Fig. 1. First, a conductive layer of W film with a thickness of 100 nm was deposited on half of a SiO_2_ wafer (thickness ~ 1 mm) through magnetron sputtering (Film, QH-1A model). Second, the dielectric layer of MgF_2_ with different periodic holes was fabricated by EBL step by step. A negative electron beam photoresist (AR-N 7520.11, Allresist) was spin-coated (60 s at 4000 r.p.m.) on the W/SiO_2_ substrate, followed by baking (90 s at 90 ℃) on a hotplate. EBL (Tescan Mira3) was performed with 30 kV accelerating voltage, 260 pA beam current, 100 μC cm^-2^ dose. After development (stirring in tetramethylammonium hydroxide (TMAH) aqueous solution with normality of 0.24 for 60 s) and rinse in DI water for 30 s, the 30 nm MgF_2_ thin film as an insulating layer was deposited on the substrate through electron-gun evaporation (FU-20PEB-RH). Then ultrasonic cleaning and an oxygen plasma treatment (100 W, 2 min) were employed to remove the resist fully. After obtaining the nanostructured anode, the handmade planar battery was assembled in Ar-filled glove box (H_2_O<0.01 ppm, O_2_<0.01ppm) by combining 3 components: nanostructure anode as the display panel on one side of the SiO_2_ substrate, LiFePO_4_ cathode (MTI cooperation) on the other side of the SiO_2_ substrate and liquid electrolyte (1.0 M lithium bis(trifluoromethanesulfonyl)imide in 1:1 vol/vol 1,3-dioxolane: dimethoxyethane with 1 wt% LiNO_3_, DodoChem Corporation). The battery was covered by another transparent glass and sealed with epoxy resin.
2. **Chromaticity calculation.** We calculated the color of the deposited Li metal hemispheres according to the International Commission on Illumination (CIE) “standard observer” functions based on human data, denoted *x*(*λ*), *y*(*λ*) and *z*(*λ*). The chromaticity coordinates are acquired by calculating the tristimulus values [1,2]:

$X=\int_{380}^{780} R(\lambda)x(\lambda)d\lambda$ (1)

$Y=\int_{380}^{780} R(\lambda)y(\lambda)d\lambda$ (2)

$Z=\int_{380}^{780} R(\lambda)z(\lambda)d\lambda$ (3)

Here *R* is the reflectivity. The integral range 380 to 780 nm represents the eye-sensitive region. Note that three tristimulus values are calculated for each reflectivity spectrum. To get the coordinates in the CIE diagram, the tristimulus values are normalized:

$\overline{x}=\frac{X}{X+Y+Z}$ (4)

$\overline{y}=\frac{Y}{X+Y+Z}$ (5)

$\overline{z}=\frac{Z}{X+Y+Z}=1-\overline{x}-\overline{y}$ (6)

To obtain the simulated colors, we used the tristimulus values under CIE standard illumination (D50/2°), and Fig. 1c shows the simulated colors of certain coordinates.

1. **Energy consumption calculation.** The coulombic efficiency (CE) is the ratio of the discharged capacity *Q_d_* to the charged capacity *Q_c_*:

$CE=\frac{Q_{d}}{Q_{c}}=\frac{\int_{0}^{t_{d}} I_{d}dt}{\int_{0}^{t_{c}} I_{c}dt}=\frac{I_{d}t_{d}}{I_{c}t_{c}}$ (7)

In this equation, *I_d_* / *I_c_* are the discharge / charge current and *t_d_* / *t_c_* are the respective discharge / charge times.

The energy efficiency (EE) is defined as the ratio of the discharged energy *E_d_* to the charged energy *E_c_*:

$EE=\frac{E_{d}}{E_{c}}=\frac{\int_{0}^{t_{d}} U_{d}I_{d}dt}{\int_{0}^{t_{c}} U_{c}I_{c}dt}$ (8)

In this equation, *I_d_* / *I_c_* are the discharge / charge current and *t_d_* / *t_c_* are the respective discharge / charge times, and *U_d_* / *U_c_* are the discharge / charge voltages. [3]

The energy consumption of our plasmonic display during the active configuration is calculated with the following formulation:

$P=\overline{U_{c}}I_{c}(1-EE)$ (9)

Here, the display device is always in the color changing state, so the charging process always exists. Here, the average charge voltage $\bar{\text{U}_{\text{c}}}\text{=3.6 V}$, $\text{I}_{\text{c}}\text{=0.25 mA }\text{cm}^{\text{-2}}$, $EE\text{≈}$56.70%, so $P\text{=0.390 mW }\text{cm}^{\text{-2}}$. If in the future $EE\text{≈ }$99.00%, $P\text{=0.009 mW }\text{cm}^{\text{-2}}\text{.}$

If the display device is in a static color state, energy consumption is calculated by:

*$P=U\cdot I$*  (10)

Here, we apply a constant voltage of *U* = 3.5 V, and the tested current is about *I* = 0.03 mA cm^-2^ shown in Supplementary Fig. 9. So the energy consumption is about 0.105 mW cm^-2^. Note: the energy efficiency was not taken into consideration.

**S. II.** **Rational designs for electrodeposition and structural transformation.**


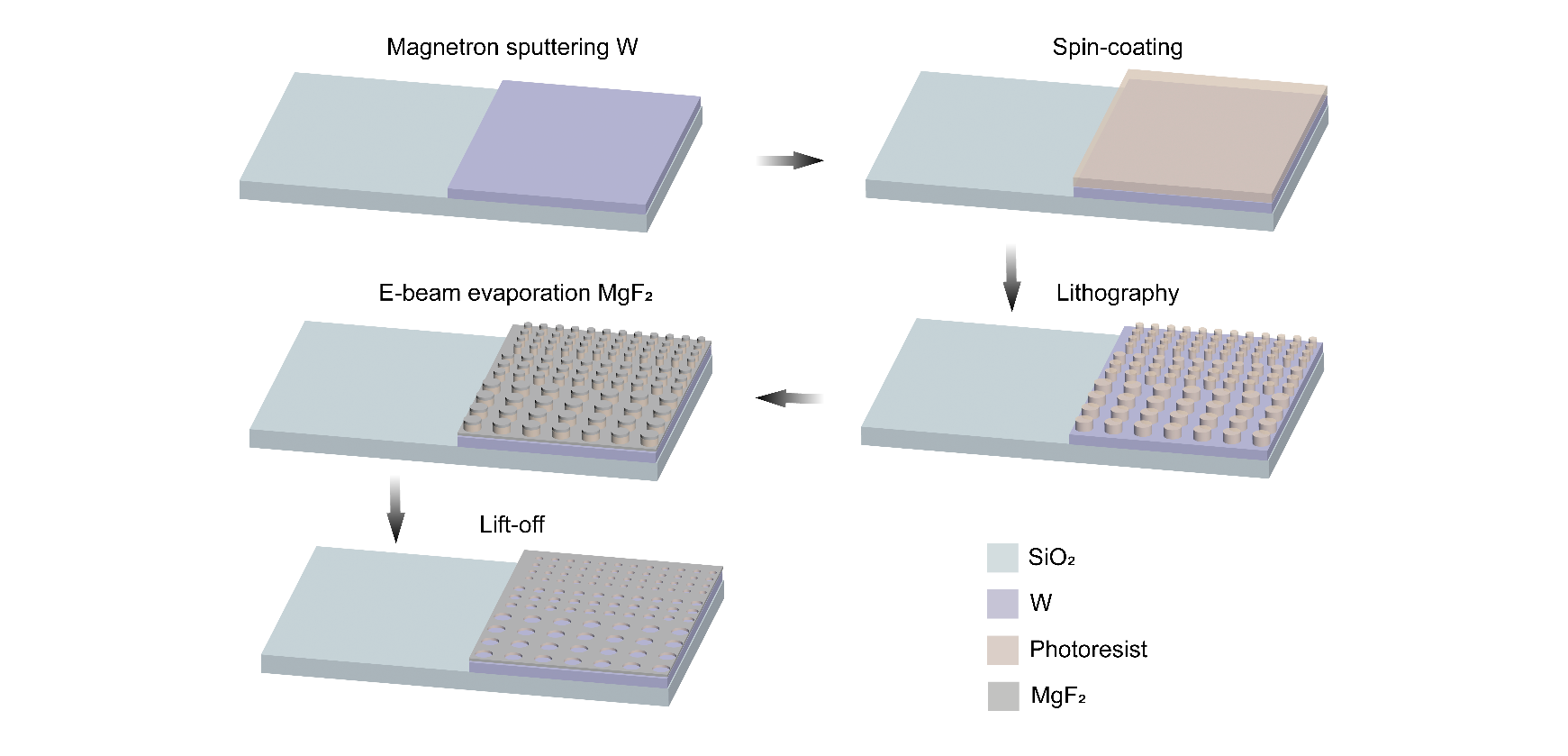


**Supplementary Fig. 1.** **The schematic of the fabrication process for the nanostructured anode.**


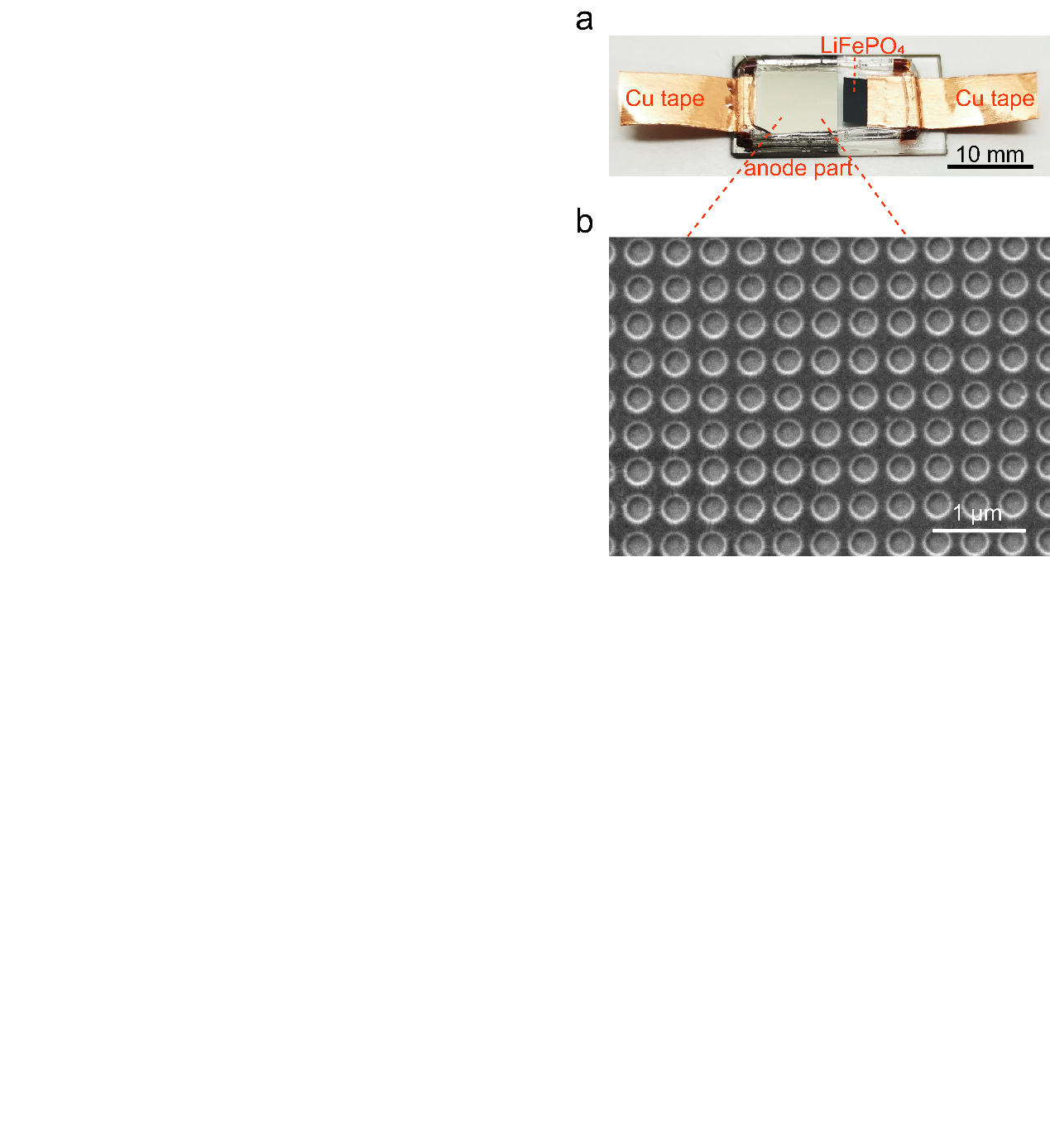


**Supplementary Fig. 2.** **(a) The photograph of the dynamic plasmonic color display: the anode part is a W film covered by the MgF_2_ layer with nanoholes for display, the cathode part is LiFePO_4_ as the Li source, and both parts are stuck to Cu tape for electrical contact. Both cathode and anode parts are immersed in the electrolyte and sealed with epoxy resin. (b) SEM image of the anode part with nanostructures.**

**S. III Optical modes of the lithium plasmonic nanostructures.**


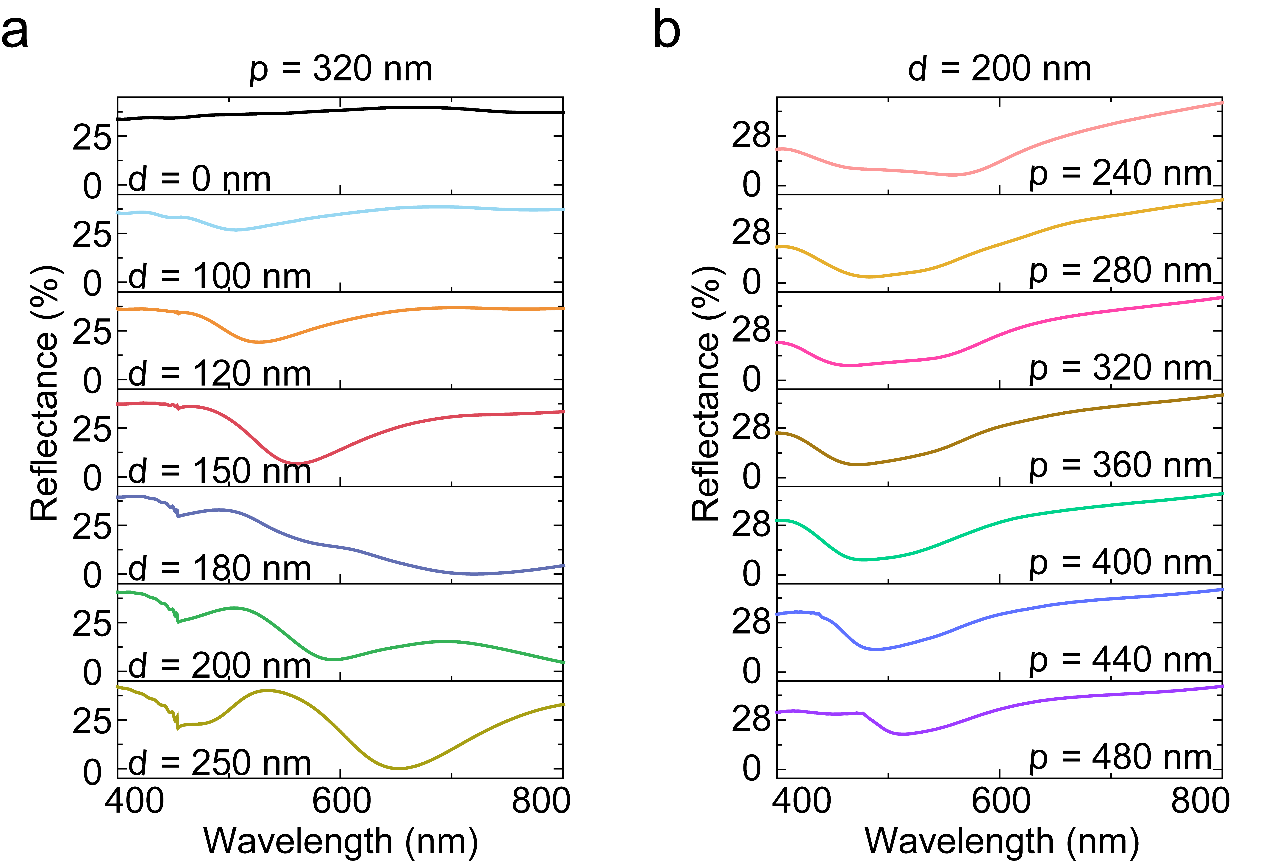


**Supplementary Fig. 3.** **(a) The simulated reflection spectra of different lithium nanoparticle sizes at a fixed period of 320 nm (*d*: 0-250 nm, reflectance valley: 500-650 nm). (b) The simulated reflection spectra of different periods at a fixed lithium nanoparticle size of 200 nm diameter (*p*: 240-480 nm, reflectance valley: 560 to 480 nm). These results indicate that LSPR dominates the plasmonic color generation.**


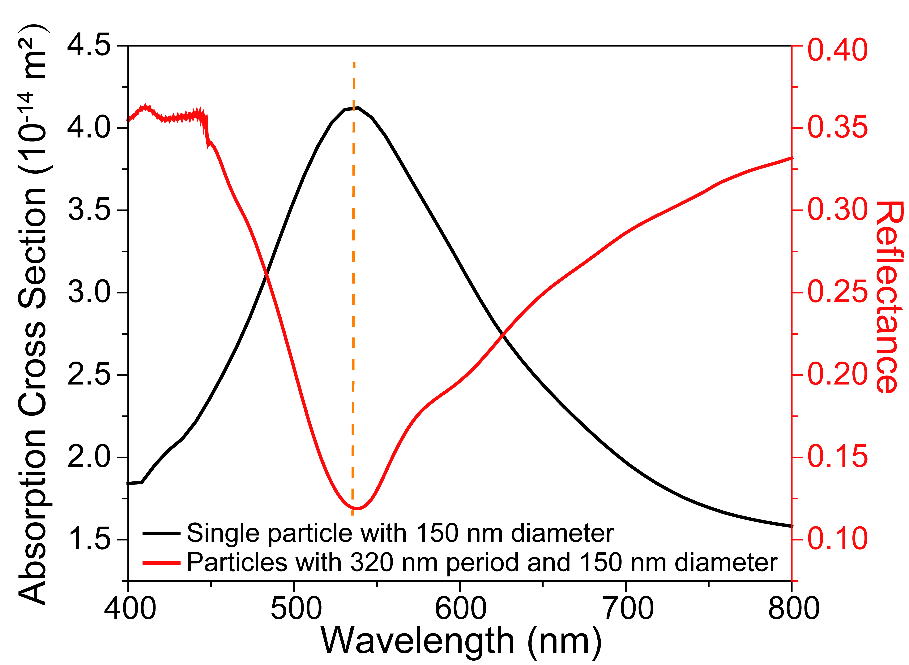


**Supplementary Fig. 4.** **The comparison of the simulated absorption cross section of a single lithium particle with 150 nm diameter and the simulated reflection spectrum of periodic lithium particles with 320 nm period and 150 nm diameter, demonstrating the LSPR mode plays a crucial role in the Li-based plasmonic color.**

**
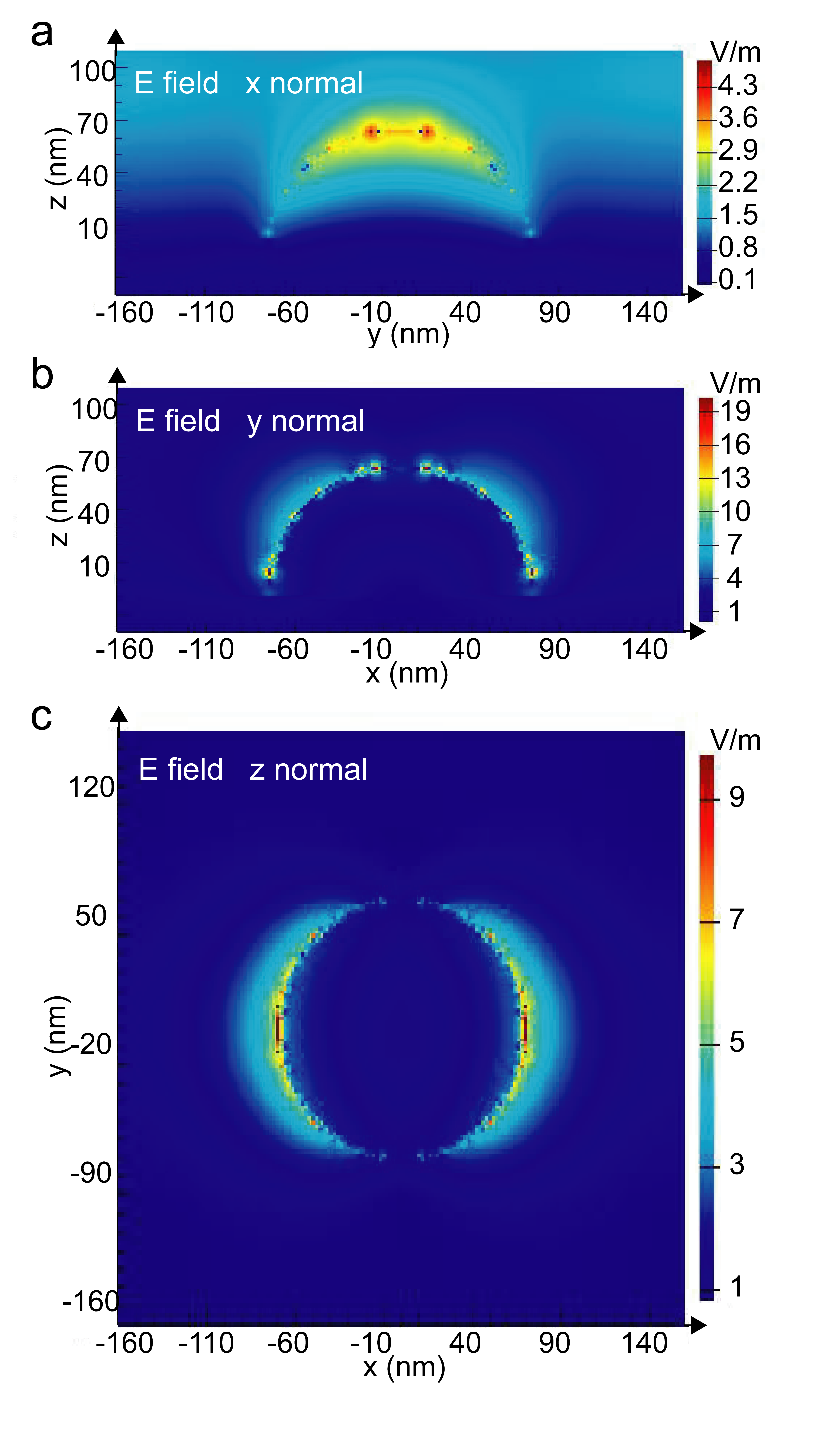
**

**Supplementary Fig. 5.** **The simulated electric field distribution of the lithium nanoparticles pattern (320 nm period and 150 nm diameter) at 535 nm wavelength, showing the LSPR properties.**


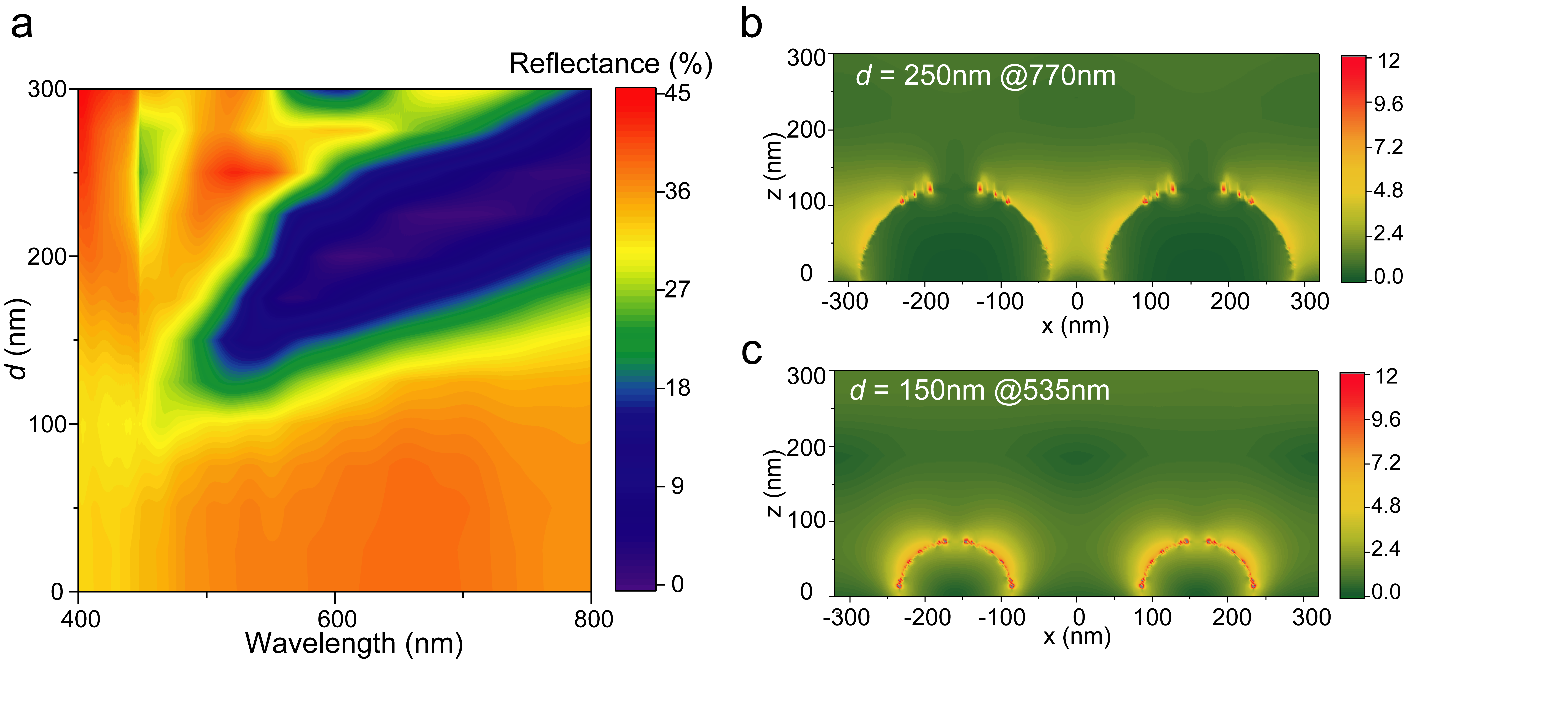


**Supplementary Fig. 6. (a) The reflectance spectra simulation of Li nanostructures of *p*=320 nm with different d (diameter). (b) The electric field distribution of the x-z plane for the nanostructure *p*=320 nm *d*=250 nm at the reflection dip wavelength 770 nm. (c) The electric field distribution of the x-z plane for the nanostructure *p*=320 nm *d*=150 nm at the reflection dip wavelength of 535 nm.** In figure a, there is an obvious reflection dip that red shifts and gradually broadens as *d* increases. When the *d* (diameter) is much smaller than *p* and the dip is narrow, for example *d*=150 nm, the electric field localized around the nanoparticles can’t affect each other (shown in figure c), which means the LSPR plays the dominant role. But as *d* gets larger, the spacing between adjacent particles decreases, and the electric field localized around the nanoparticles can affect each other (shown in figure b) and a hybrid plasmon resonance mode generates, so the reflection dip broadens. And when *d* > 200 nm, a slight dip at around 448 nm appears which is constant and has nothing with the particle size, so the new appearing dip may ought to the hybrid SPP mode.


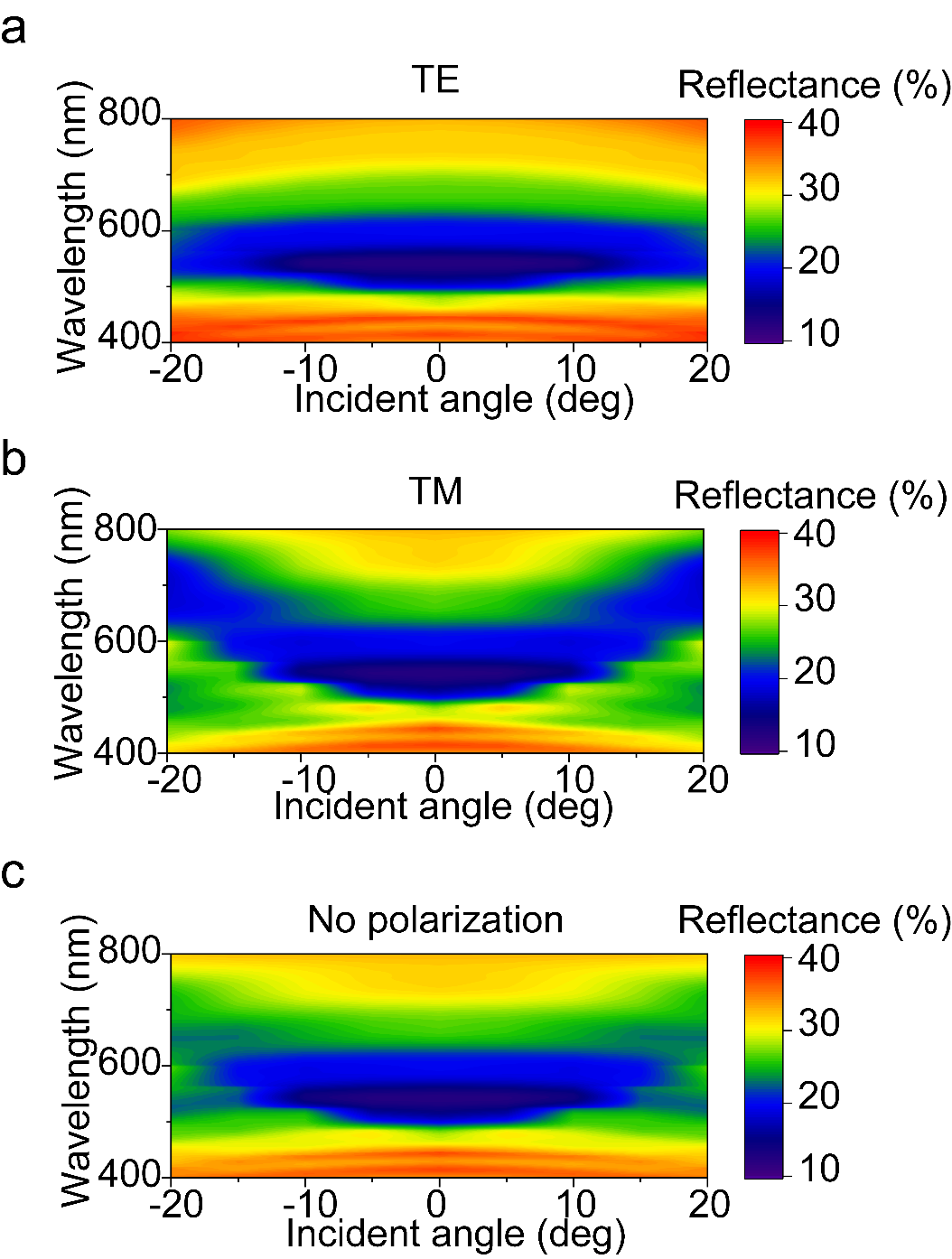


**Supplementary Fig.7. The simulation of angularly resolved reflectance spectra of nanostructures of *p*=320 nm, *h*=150 nm and *d*=150 nm under TE illumination (a), TM illumination (b) and no polarized illumination.**

**S. IV Plasmonic color generation in the charging/discharging processes.**


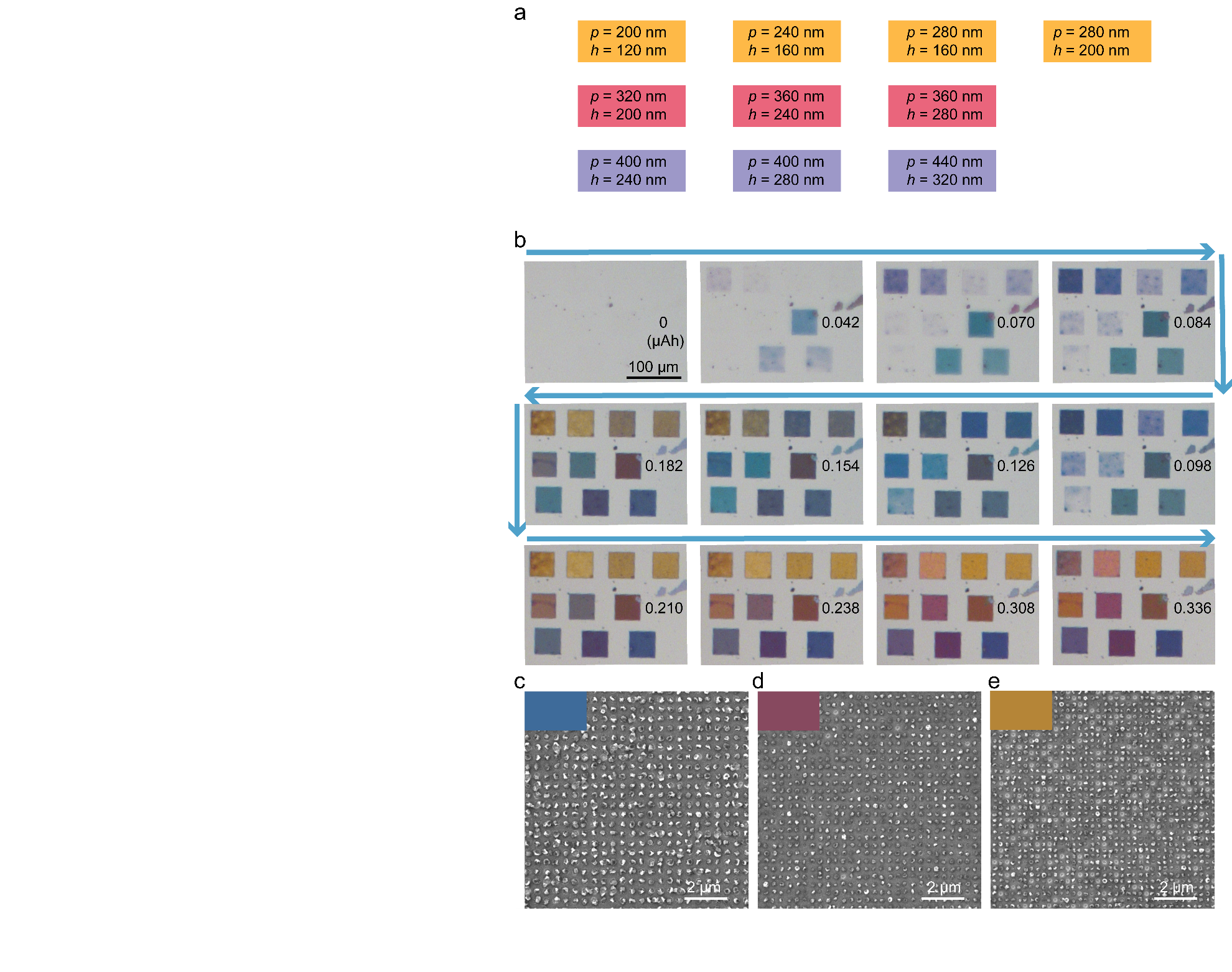


**Supplementary Fig. 8.** **(a) The designed patterns of different nanostructures. (b) Optical images of the different colors evolution of different patterns during the charging process. (c-e) The SEM images after charging of the blue pattern *p* = 440 nm, *d* = 280 nm (c), red pattern *p* = 360 nm, *d* = 240 nm (d) and yellow pattern *p* = 280 nm, *d* = 160 nm (e).** **The specific color and color generation speed depend on geometry parameters (*p* and *h*) and charging capacity.**


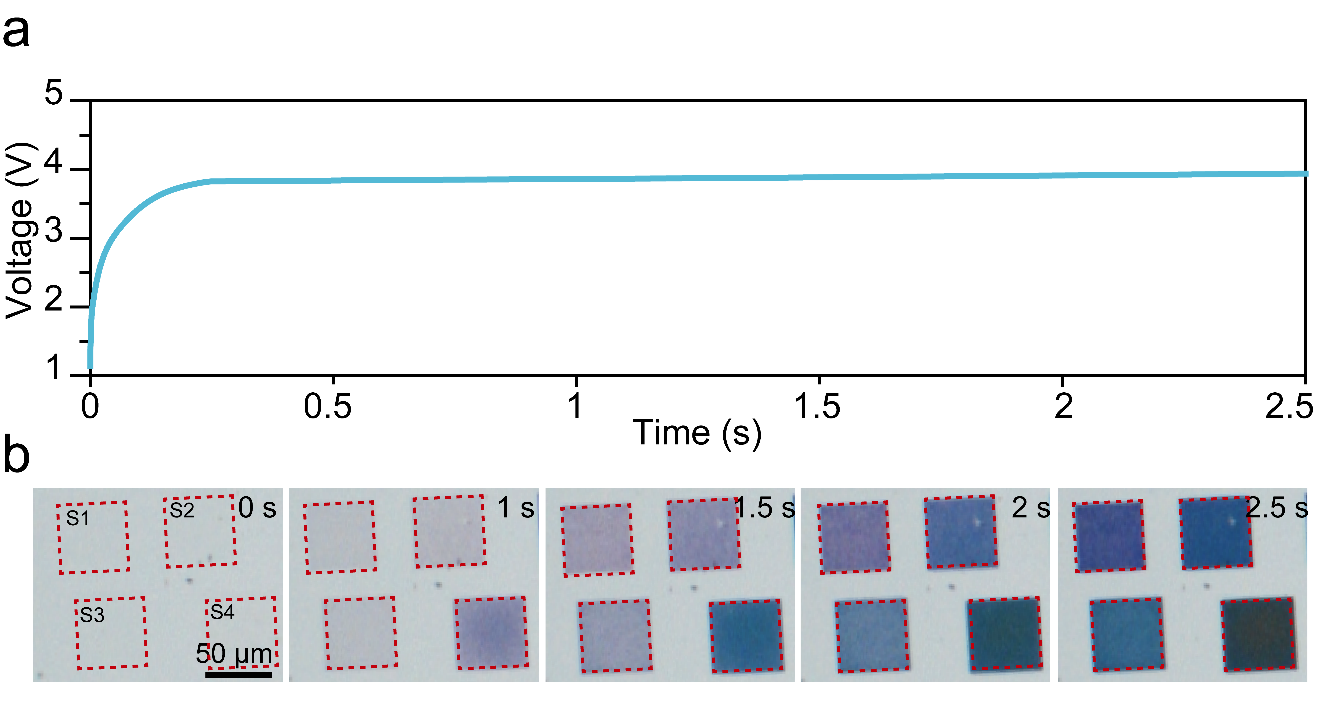


**Supplementary Fig. 9. (a) The electrochemical voltage-time curve of fast plating for 2.5 s. (b) The color evolution images for the fast plating showing the uniform color generation of 4 different patterns: *p*=320 nm *h*=200 nm for S1, *p*=360 nm *h*=240 nm for S2, *p*=400 nm *h*=240 nm for S3, *p*=400 nm *h*=280 nm for S4.**


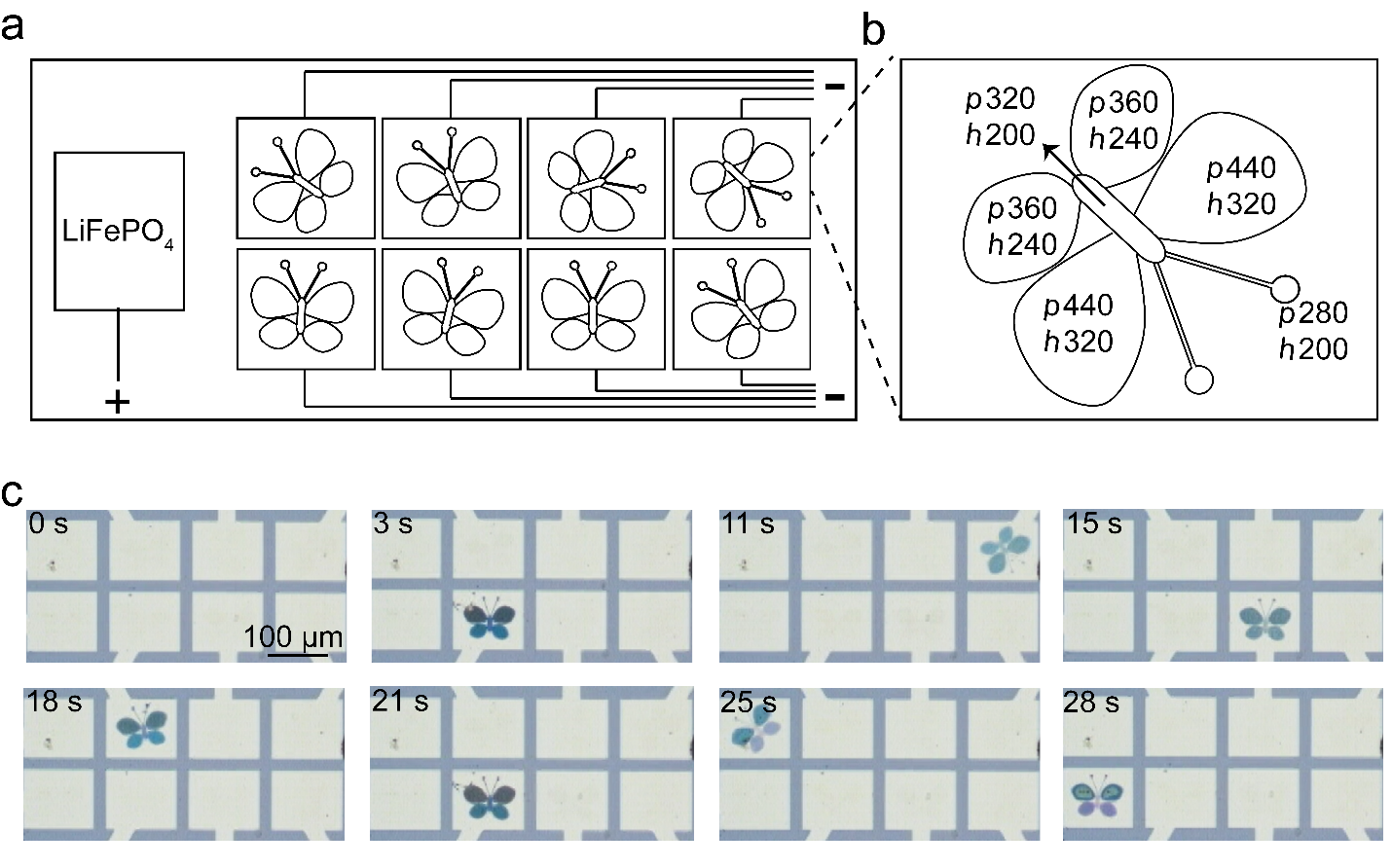


**Supplementary Fig. 10.** **Micro-animation of a flying butterfly. (a) Schematic of a flying butterfly device: the display panel is composed of 8 independent patterns. (b) Design of a butterfly with geometry parameters. (c) The dynamic process of the plasmonic micro-animation of a flying butterfly over time. The flying butterfly was obtained by the continuous charging (butterfly generation) and discharging (butterfly erasure) processes of each selected independent pattern.**

**S. V Electrochemical properties of the full-cycle display process.**


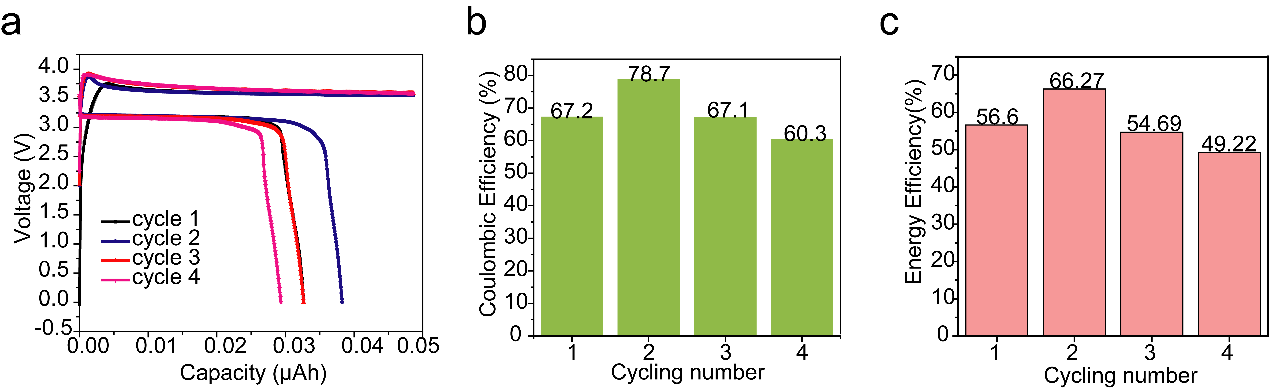


**Supplementary Fig. 11.** **Electrochemical performance of the battery for the blue color display. (a) The charging and discharging curves for different cycles. The applied charging/discharging current density are about 0.25 mA cm^-2^ and 0.125 mA cm^-2^, respectively. (b) The coulombic efficiencies for 4 cycles. (c) The energy efficiency for 4 cycles.**


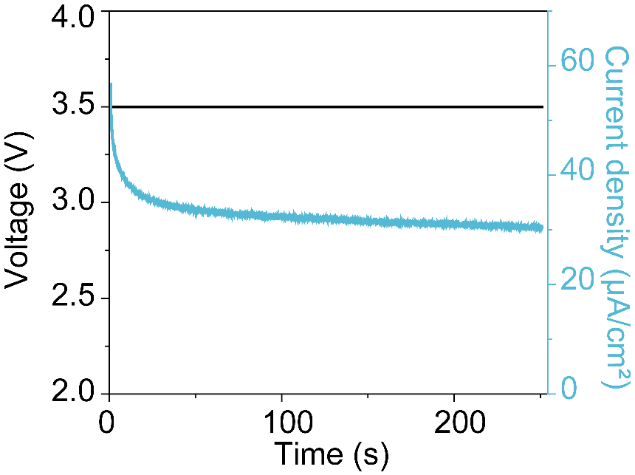


**Supplementary Fig. 12.** **The current profile while applying a constant voltage (3.5 V) to maintain the static color.**


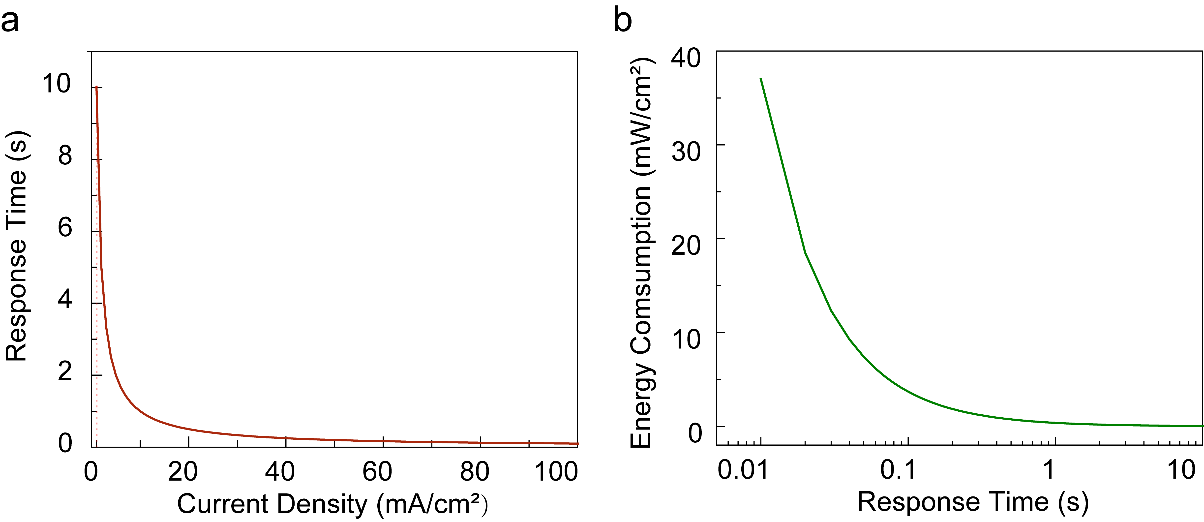


**Supplementary Fig. 13. The** **prospective display performance of response time and corresponding current density and energy consumption.**

**S. VI On-chip demonstration of the low-powered plasmonic color display panel.**


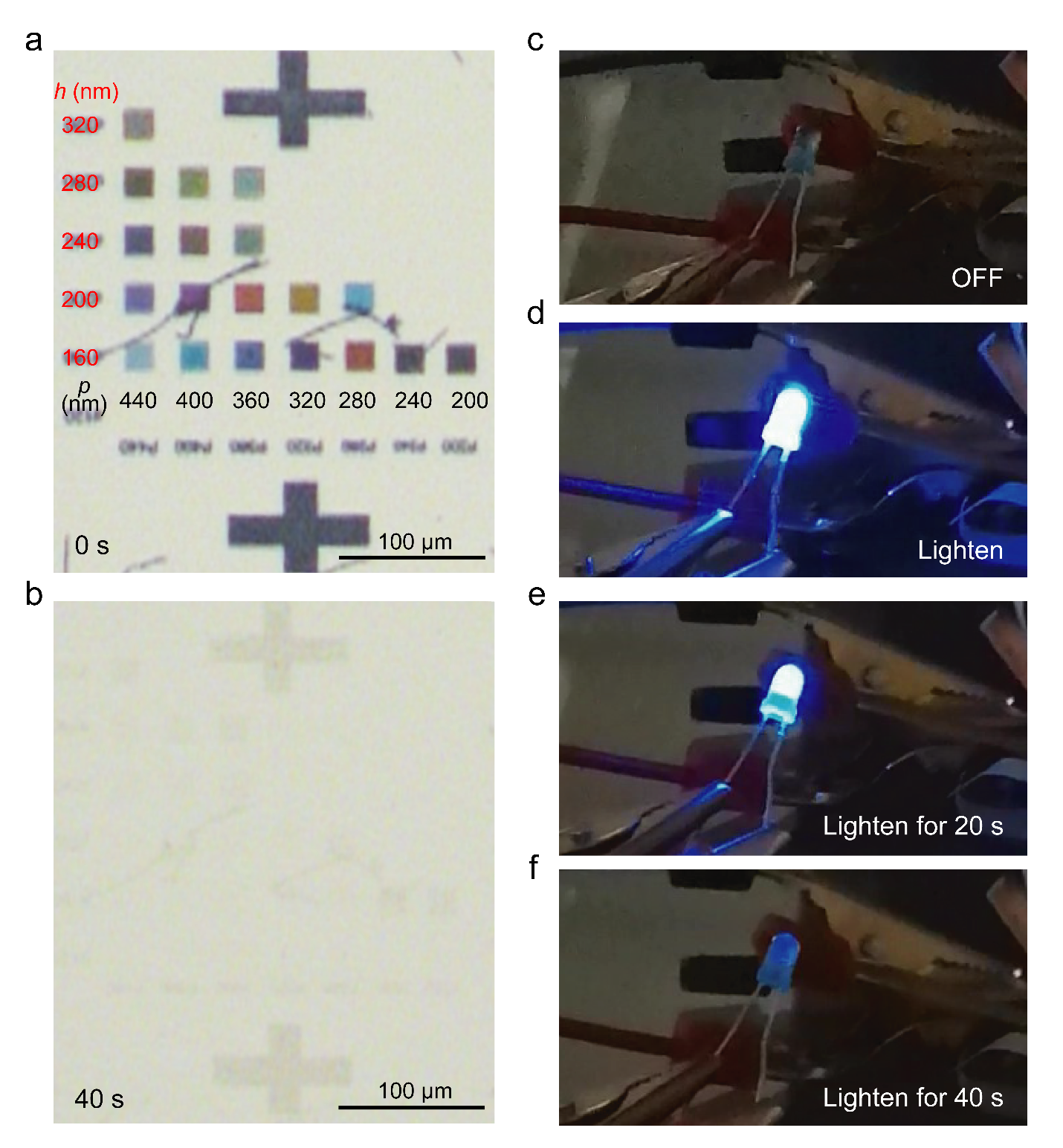


**Supplementary Fig. 14.** **Energy storage performance of the integrated display chip: lighting a blue LED for 40 s. (a) The pre-charged colored display chip at 0 s. (b) The erased display chip after lighting a blue LED for 40 s. (c-f) Pictures of the blue LED at different statuses. The traces in (a) due to fabrication flaws are also erased in (b).**

**
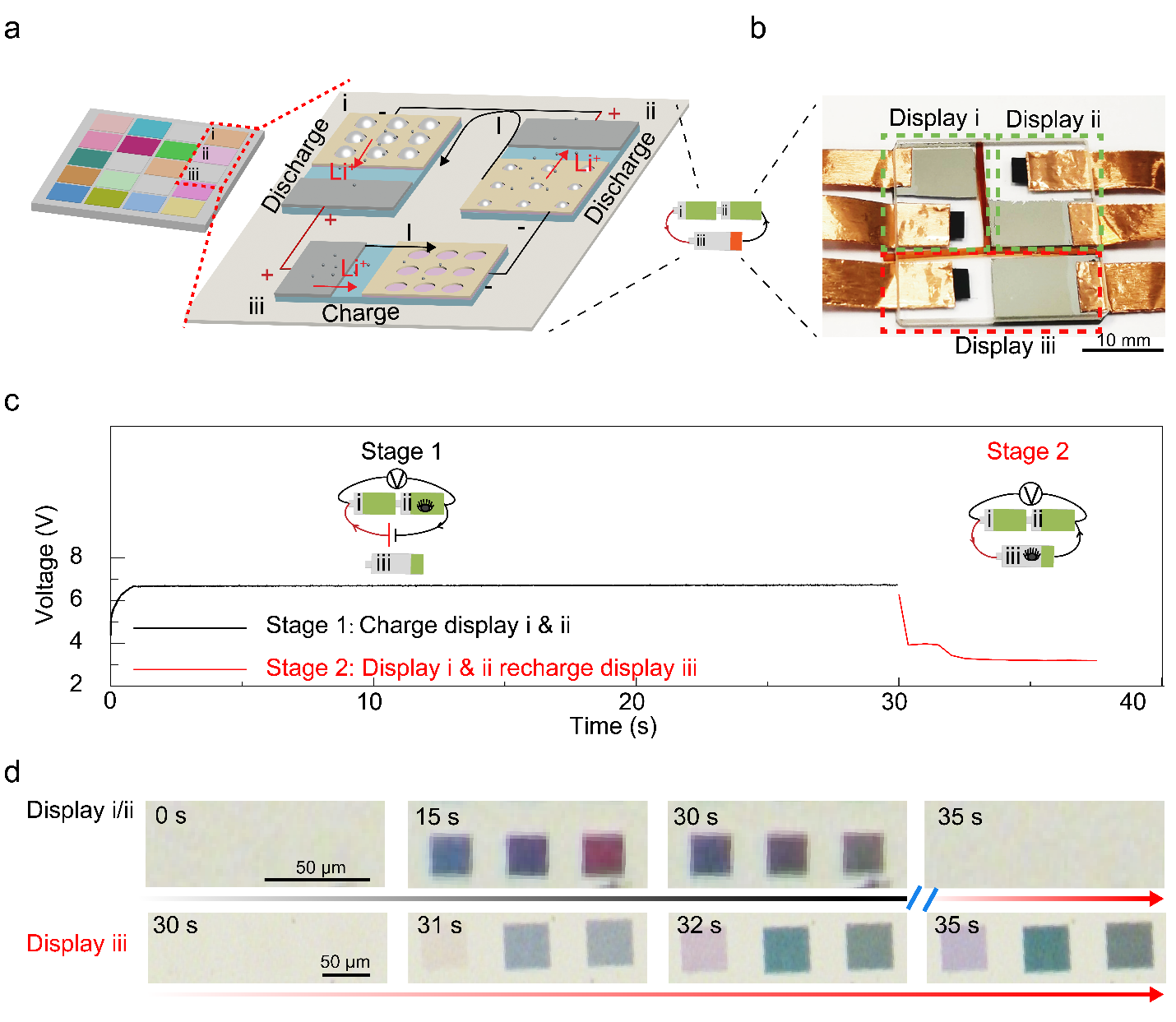
**

**Supplementary Fig. 15.** **The low-powered dynamic plasmonic display. (a-b) The schematic and optical image of the low-powered dynamic plasmonic color device. (c) The experimental voltage-time curve of the low-powered dynamic plasmonic color display. Insets are the schematic diagrams of devices in different experimental statuses. (d) The corresponding experimental color evolution of the display i or ii and display iii.** Firstly, the tandem display panels (i and ii) are pre-charged for color generation for 30 s (Stage 1) at around 0.25 mA cm^-2^; and then, the tandem display panels (i and ii) recharge towards the third display panel (iii) from 30 s to 35 s for color generation of the third display panel (iii) and color erasing of the tandem display panels (i and ii) (Stage 2). During stage 2, colors can be erased (display i and ii) and generated (display iii) without external power input, unraveling the self-power nature of the overall device. Moreover, in the future, we can also design proper circuits to achieve DC-DC voltage increasing conversion to realize one pixel panel-power-one pixel panel.


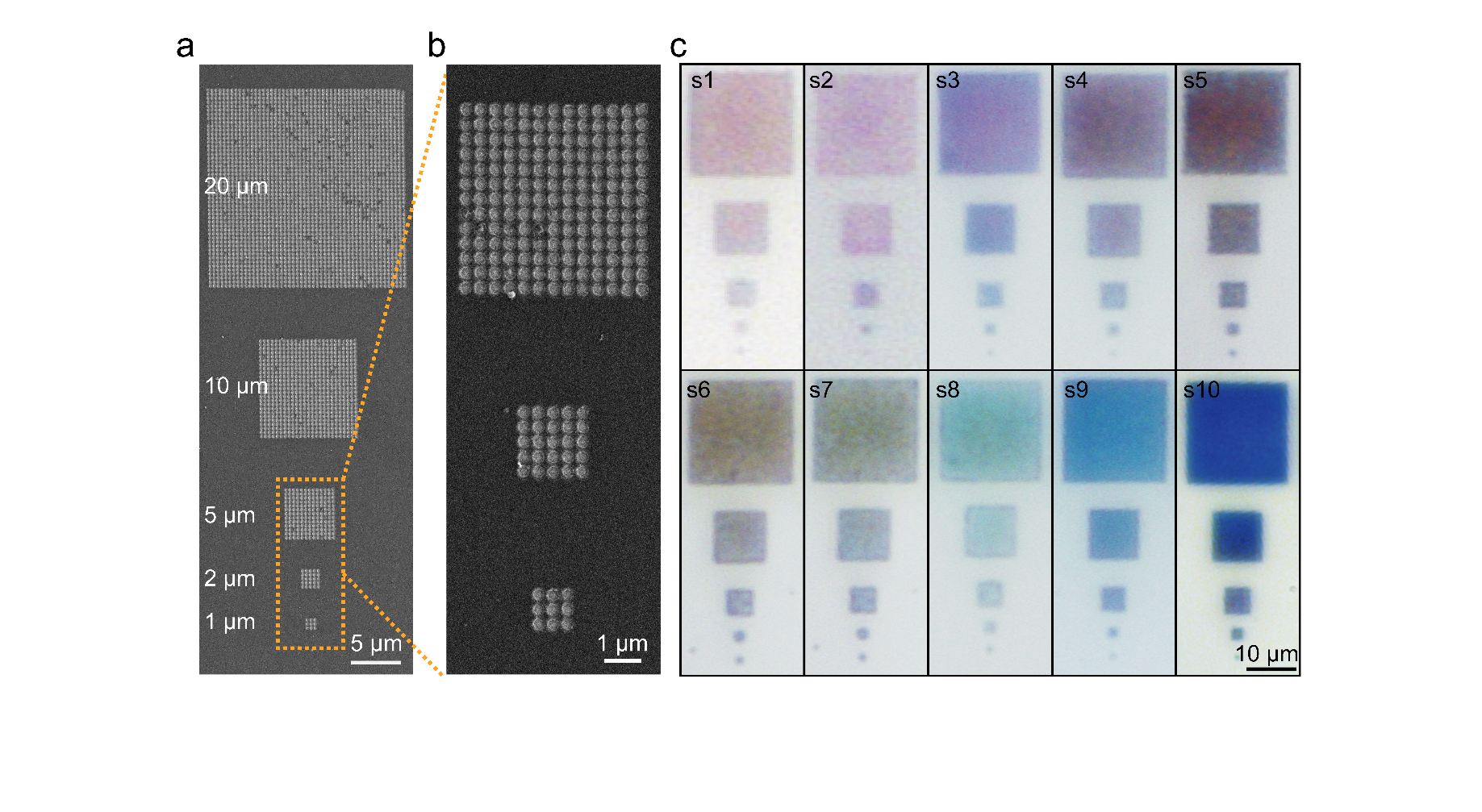


**Supplementary Fig. 16. High resolution performance of the plasmonic color display. (a-b) SEM images of pixels with different sizes (20 μm, 10 μm, 5 μm, 2 μm, 1 μm). In each pixel, *p*=400 nm, *h*=280 nm. (c) Optical images of different size pixels and different colors (s1, s2: *p*=360 nm, *h*=280 nm; s3, s4, s5: *p*=320 nm, *h*=200 nm; s6, s7: *p*=280 nm, *h*=200 nm; s8, s9, s10: *p*=400 nm, *h*=240 nm).**

**References**

1. Xiong K, Emilsson G and Maziz A *et al.* Plasmonic Metasurfaces with conjugated polymers for flexible electronic paper in color. *Adv. Mater.*2016; **28**: 9956-9960.

2. Duan X, Kamin S and Liu N. Dynamic plasmonic colour display. *Nat. Commun.* 2017; **8**: 14606.

3. Meister, P, Jia HP and Li J *et al*. Best practice: performance and cost evaluation of lithium ion battery active materials with special emphasis on energy efficiency. *Chem. Mater.* 2016; **28**: 7203-7217.
